# Comparison Analysis of Dysregulated LncRNA Profile in Mouse Plasma and Liver after Hepatic Ischemia/Reperfusion Injury

**DOI:** 10.1371/journal.pone.0133462

**Published:** 2015-07-29

**Authors:** Zhenzhen Chen, Yanjin Luo, Weili Yang, Liwei Ding, Junpei Wang, Jian Tu, Bin Geng, Qinghua Cui, Jichun Yang

**Affiliations:** 1 Department of Physiology and Pathophysiology, Peking University School of Basic Medical Sciences, 38 Xueyuan Road, Beijing, 100191, China; 2 Department of Biomedical Informatics, Peking University School of Basic Medical Sciences, 38 Xueyuan Road, Beijing, 100191, China; 3 Institute of Systems Biomedicine, Peking University, 38 Xueyuan Road, Beijing, 100191, China; 4 MOE Key Lab of Molecular Cardiovascular Science, Peking University, 38 Xueyuan Road, Beijing, 100191, China; 5 Center for Noncoding RNA Medicine, Peking University Health Science Center, Beijing, 100191, China; IDIBAPS - Hospital Clinic de Barcelona, SPAIN

## Abstract

Long noncoding RNAs (LncRNAs) have been believed to be the major transcripts in various tissues and organs, and may play important roles in regulation of many biological processes. The current study determined the LncRNA profile in mouse plasma after liver ischemia/reperfusion injury (IRI) using microarray technology. Microarray assays revealed that 64 LncRNAs were upregulated, and 244 LncRNAs were downregulated in the plasma of liver IRI mouse. Among these dysregulated plasma LncRNAs, 59-61% were intergenic, 22-25% were antisense overlap, 8-12% were sense overlap and 6-7% were bidirectional. Ten dysregulated plasma LncRNAs were validated by quantitative PCR assays, confirming the accuracy of microarray analysis result. Comparison analysis between dysregulated plasma and liver LncRNA profile after liver IRI revealed that among the 308 dysregulated plasma LncRNAs, 245 LncRNAs were present in the liver, but remained unchanged. In contrast, among the 98 dysregulated liver LncRNAs after IRI, only 19 were present in the plasma, but remained unchanged. LncRNA AK139328 had been previously reported to be upregulated in the liver after IRI, and silencing of hepatic AK139328 ameliorated liver IRI. Both microarray and RT-PCR analyses failed to detect the presence of AK139328 in mouse plasma. In summary, the current study compared the difference between dysregulated LncRNA profile in mouse plasma and liver after liver IRI, and suggested that a group of dysregulated plasma LncRNAs have the potential of becoming novel biomarkers for evaluation of ischemic liver injury.

## Introduction

About 5% to 10% of the human genome sequence was transcripted. Among human transcripts, it is estimated that the protein-coding RNAs only account for about 10–20%, and the remaining 80% ~ 90% were non-protein-coding transcripts, which were termed as non-coding RNAs [1, 2]. Long non-coding RNA (LncRNA) are a class of noncoding RNAs with the length greater than 200nt and consists of the majority of non-coding RNAs [3]. Based on their relative position in the genome to the protein-coding genes, LncRNAs can be divided into five categories: sense, antisense, intronic, intergenic and bidirectional [4]. LncRNAs are widely involved in various physiological and pathophysiological processes by functioning as cis-tether, cis-targeting, trans-targeting, enhancer, decoy, scaffold, allosteric modification, co-activator or co-repressor to modulate gene expression in various cell types [5]. Moreover, not only LncRNAs in the tissue, but also those in circulation are shown to be widely involved in the pathogenesis of various diseases [6]. The use of tissue samples for detection of biomarker molecules has significant limitations such as limited sample sources, safety, unable to continue testing and follow-up. Therefore, analyzing LncRNAs in circulation represents promising and non-invasive methods for the detection and diagnosis of various diseases. LncRNA-PCA3 expression in prostate cancer tissues was significantly higher than that of adjacent tissues. Further studies have shown that LncRNA-PCA3 is specifically expressed in prostate tissue in human [7]. So far, detection of LncRNA-PCA3 level in the urine has been approved by the US Food and Drug Administration to facilitate the diagnosis prognosis of prostate cancer in clinical trials.

Interruption of blood flow or lack of oxygen leads to hepatic ischemia, and hepatic ischemia-reperfusion injury (IRI) is a phenomenon whereby hepatocyte dysfunction and damage happens when the reestablishment of blood supply in ischemic liver [8]. In some severe cases, liver IRI may lead to liver congestion, progressive thrombosis and organ necrosis [8]. At present, the main effective measure for ameliorating liver IRI is ischemic preconditioning (IPC). Some certain medicines such as vitamin E and glutamine have also been shown to enhance the tolerance of hepatocytes to IRI [9]. To date, several studies have revealed the important roles of dysregulated LncRNA profiles in the pathogenesis of ischemic injury in various organs including liver, heart and brain [10–14]. These findings strongly suggested that LncRNAs in tissues are widely involved in the progression of diseases. However, the LncRNA profile in the plasma of liver IRI still remains unknown. Determination of the dysregulated LncRNA profile in the plasma will not only be very helpful for further understanding the pathogenesis of liver IRI, but also provide insight into identifying some LncRNAs as potential biomarkers for assessing the severity of ischemic liver damage.

In the current study, the LncRNA expression profile in the plasma of mice after hepatic IRI was determined using microarray technology, and further compared with that in liver reported in our previous study [12]. The comparison analysis results revealed that most of the dysregulated plasma LncRNAs may come from other tissues or cells beyond liver cells after liver IRI. Overall, these findings suggested that analysis of some circulating LncRNA levels may be useful for evaluating the severity of ischemic liver diseases.

## Experimental Procedures

### Animal ethics statement

All animal care and experimental protocols complied with the Animal Management Rules of the Ministry of Health of the People’s Republic of China and the guide for the Care and Use of the Laboratory Animals of the Peking University Health Science Center. All animal protocols were approved by the Animal Research Committee of the Peking University Health Science Center that complies with the Guide for the Care and Use of Laboratory Animals published by the US National Institutes of Health (NIH Publication No. 85–23, revised 1996).

### Generation of partial liver ischemia/reperfusion injury model

Eight to ten week old male C57BL/6 mice weighing 26-28g were used in all experiments. Animals were housed in pathogen free conditions and received humane care. Mice were fed and received tap water ad libitum. The animals underwent either sham surgery or I/R treatment. The protocol for generation of a murine model of 70% partial hepatic ischemia had been detailed in our previous study [12]. Briefly, mice were anesthetized by intraperitoneal injection of sodium pentobarbital (60 mg/kg). A midline laparotomy was performed, and blood supply to the left lateral and median lobes of the liver was interrupted using an atraumatic artery clip. After 60 minutes of partial hepatic ischemia, the clip was removed to initiate hepatic reperfusion. Sham control mice underwent the same protocol without vascular occlusion. Mice were sacrificed after 6 hours of reperfusion, and then blood and samples of the left lateral lobe were taken for analysis. The sham mice were used as control and the IRI-treated mice were used as experiment group for microarray analysis and further analysis.

### Quantitative RT-PCR assays of LncRNA expression in mouse plasma

The liver and plasma were quickly isolated and immediately frozen in liquid nitrogen. Total RNA was extracted and reverse transcribed to cDNA using of the RevertAid First Strand cDNA Synthesis Kit (Thermo scientific) according to the manufacturer’s protocol using random primers as detailed previously [12].

The protocol for quantitative real-time PCR assays of LncRNAs has also been described in our previous study [12]. (The detailed information regarding the primers for analysis of LncRNA expression was provided in S1 Table). The *Gapdh* gene has been used as an internal control for analyzing plasma LncRNAs expression levels as indicated in previous study [12, 15].

### Determination of plasma LncRNA profile of ischemia/reperfusion-injured mouse

The expression profiles of LncRNAs in mouse plasma of sham and IRI groups were detected using Arraystar Mouse LncRNA Microarray v2.0 by KangChen Bio-tech (Shanghai, China) as detailed in previous studies [12, 15]. The microarray analysis was performed using a pooled plasma of 5–8 mice with liver IRI or sham operation. In our previous studies, similar microarray assays had also been performed using pooled plasma, blood, liver, heart or cell samples [12, 15, 16]. Real time PCR assays had proved the accuracy of microarray data obtained from pooled samples in these studies [12, 15, 16]. The expression profile dataset was submitted to the NCBI GEO database (GEO accession number: GSE60726). For microarray analysis, Agilent Feature Extraction software (version 11.0.1.1) was used to analyze the acquired array images. Quantile normalization and subsequent data processing were performed using the GeneSpring GX v11.5.1 software package (Agilent Technologies). After quantile normalization of the raw data, LncRNAs that at least 2 out of 2 samples have flags in Present or Marginal (“All Targets Value”) were chosen for differentially expressed LncRNAs screening. The threshold for up-regulation was fold change≥1.5 and for down-regulation, fold change≤0.7.

### Statistical analysis

Data were presented as mean ± SD. Statistical significance of differences between groups was analyzed by *t*-test or by one-way analysis of variance (ANOVA) when more than two groups were compared.

## Results

### Microarray assays of dysregulated plasma LncRNA profile after hepatic IRI

A partial hepatic I/R model of mice had been generated as detailed previously [12] to determine the plasma LncRNA profile after liver IRI.

**Fig 1:**
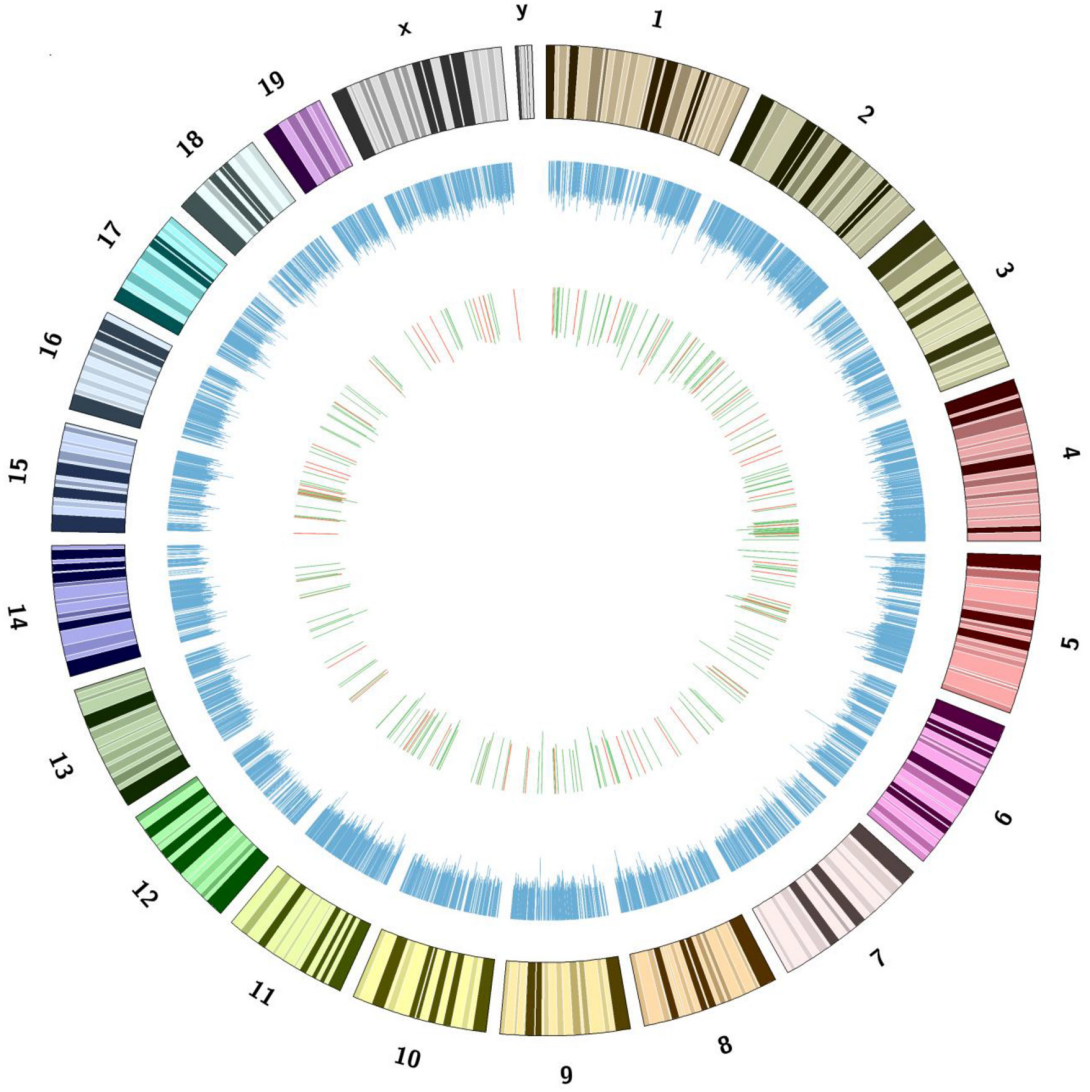
Genomic loci of the plasma LncRNAs in mouse chromosomes. The loci of LncRNAs in mouse chromosomes has been indicated in blue. The increased and decreased LncRNAs had been marked in red and green, respectively. The bars represent the folds of LncRNAs expression change. The numbers and symbols represent chromosome numbers. Please refer to GEO accession number GSE60726 for the detailed information of all plasma LncRNAs.

**Fig 2:**
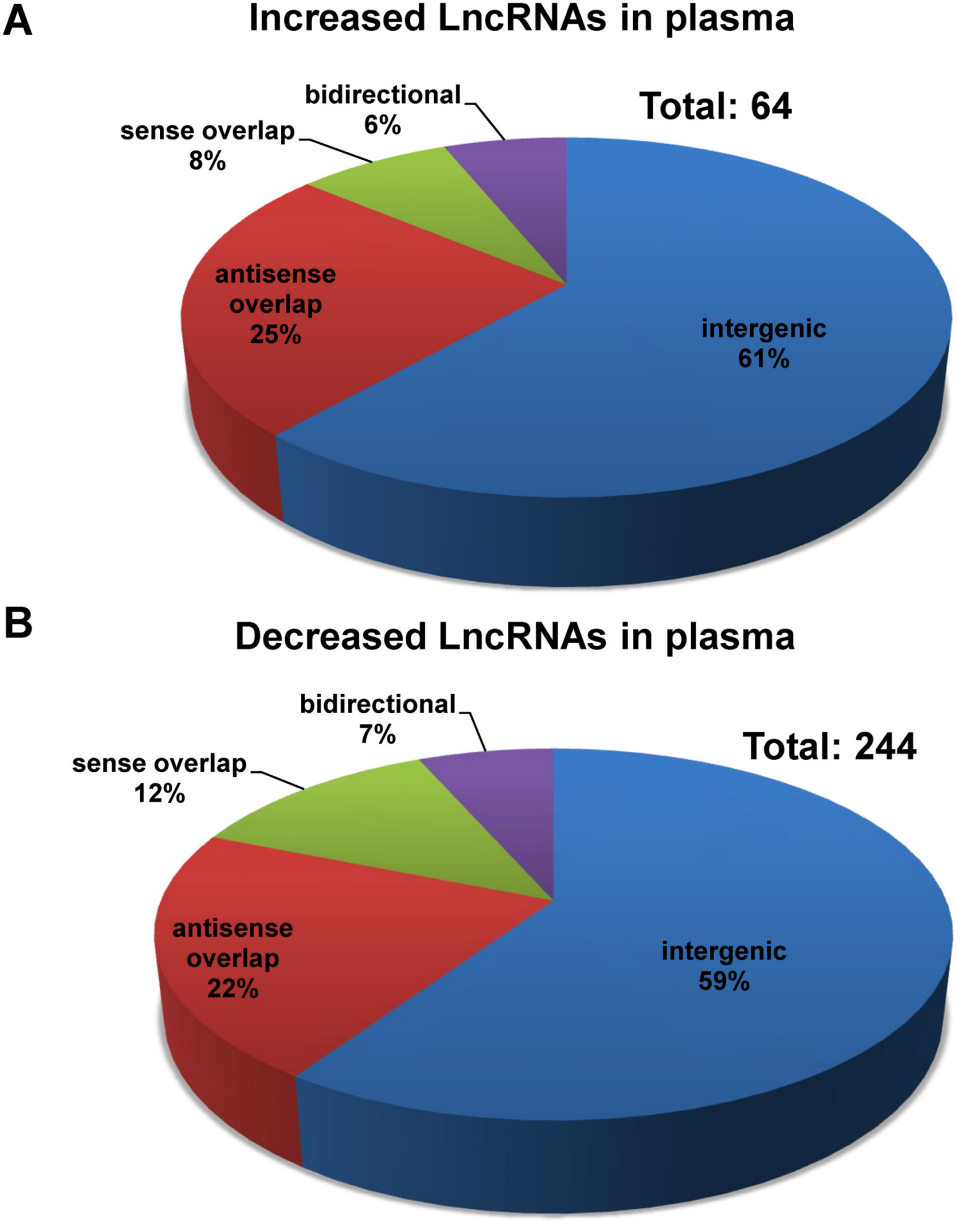
Dysregulated LncRNA profile in the plasma of I/R-treated mouse. Dysregulated LncRNAs in mouse plasma were identified by microarray assays as described in the methodology section. The threshold for up-regulation was fold change≥1.5 and for down-regulation, fold change≤0.7. Increased LncRNAs was presented in panel A, whereas decreased LncRNAs presented in panel B. The detailed information of the dysregulated LncRNAs was referred to S2 and S3 Tables.

The LncRNA profile in the plasma of mice after hepatic IRI was determined by KangChen Bio-tech (Shanghai, China) using microarray technology [12, 15, 17]. Microarray assays detected the signals of a total 10206 LncRNAs in mouse plasma (The detailed information was referred to GEO accession number: GSE60726). These plasma LncRNAs are ubiquitously distributed in all chromosomes including sex chromosomes of mouse (Fig 1). Among the plasma LncRNAs after IRI, 64 LncRNAs were upregulated (Fig 2A), whereas 244 LncRNAs were downregulated (Fig 2B) when compared with sham mice. The detailed information including name, fold of change, chromosome location, size and other characters of these dysregulated LncRNAs in mouse plasma were provided in S2 and S3 Tables. In the same tables, whether one plasma LncRNA was present in the liver or not had also been indicated in the last column in red (The detailed information regarding the LncRNA expression profile in mouse liver after IRI had been referred to the previous study [12]).

Among the dysregulated plasma LncRNAs, 59–61% were intergenic, 22–25% were antisense overlap, 8–12% were sense overlap, and 6–7% were bidirectional (Fig 2A and 2B). In contrast, among all plasma LncRNAs, 58% were intergenic, 22% were antisense overlap, 12% were sense overlap, and 7% were bidirectional. To test whether there is a potentially enrichment of specific types of LncRNAs in the plasma, we performed chi square test. The numbers of the four types of dysregulated plasma LncRNAs are 185 (intergenic), 72 (overlap), 31 (sense overlap), and 20 (bidirectional), respectively. In contrast, the numbers of the four types of non-dysregulated plasma LncRNAs are 5734, 2173, 1194 and 694, respectively (The types of a small number of plasma LncRNAs were not indicated in the original microarray data). Chi square test was further performed using the R software. The analytical results revealed that the distribution of dysregulated LncRNAs is not significantly different from that of the non-dysregulated ones (P = 0.6664). Clearly, there is not a specific type of LncRNAs enriched in the dysregulated plasma LncRNAs after liver IRI.

### Quantitative assays of dysregulated plasma LncRNAs after hepatic ischemia-reperfusion injury

**Fig 3:**
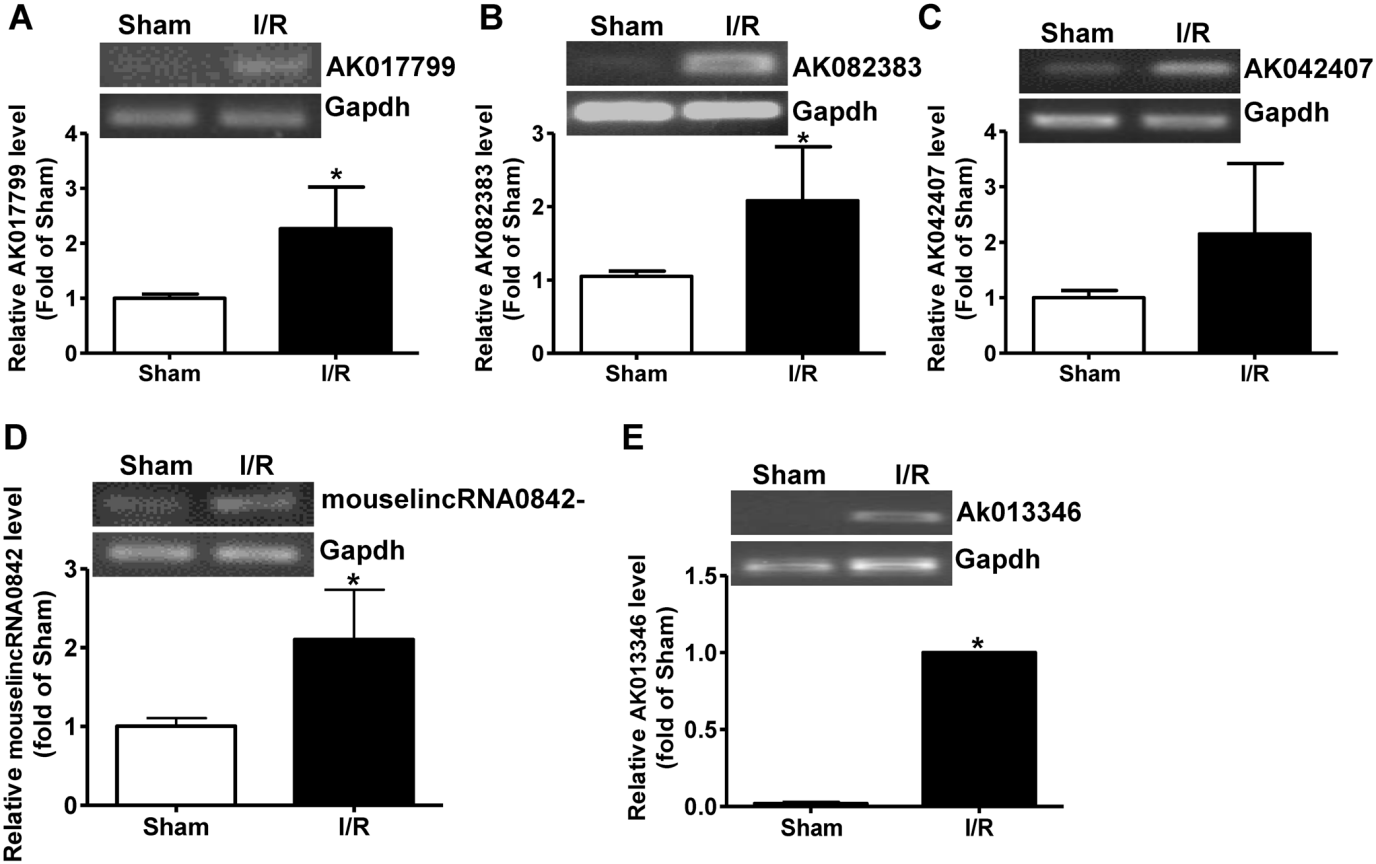
Quantitative assays of increased LncRNAs in I/R-treated mouse plasma. A-E) Five upregulated LncRNAs with most meaningful elevation in expression fold picked up by microarray assays were further analyzed and confirmed by quantitative RT-PCR assay. In panel A-D, the data were normalized to sham group. In Panel E, the data were normalized to I/R group because the signal of LncRNA Ak013346 in real time RT-PCR assays was too weak in sham group. The representative gel image had been provided for analysis of each LncRNA. I/R, ischemia/reperfusion; Sham, plasma of sham mice; I/R, plasma of I/R-treated mice. N = 5, *P<0.05 versus sham group.

**Fig 4:**
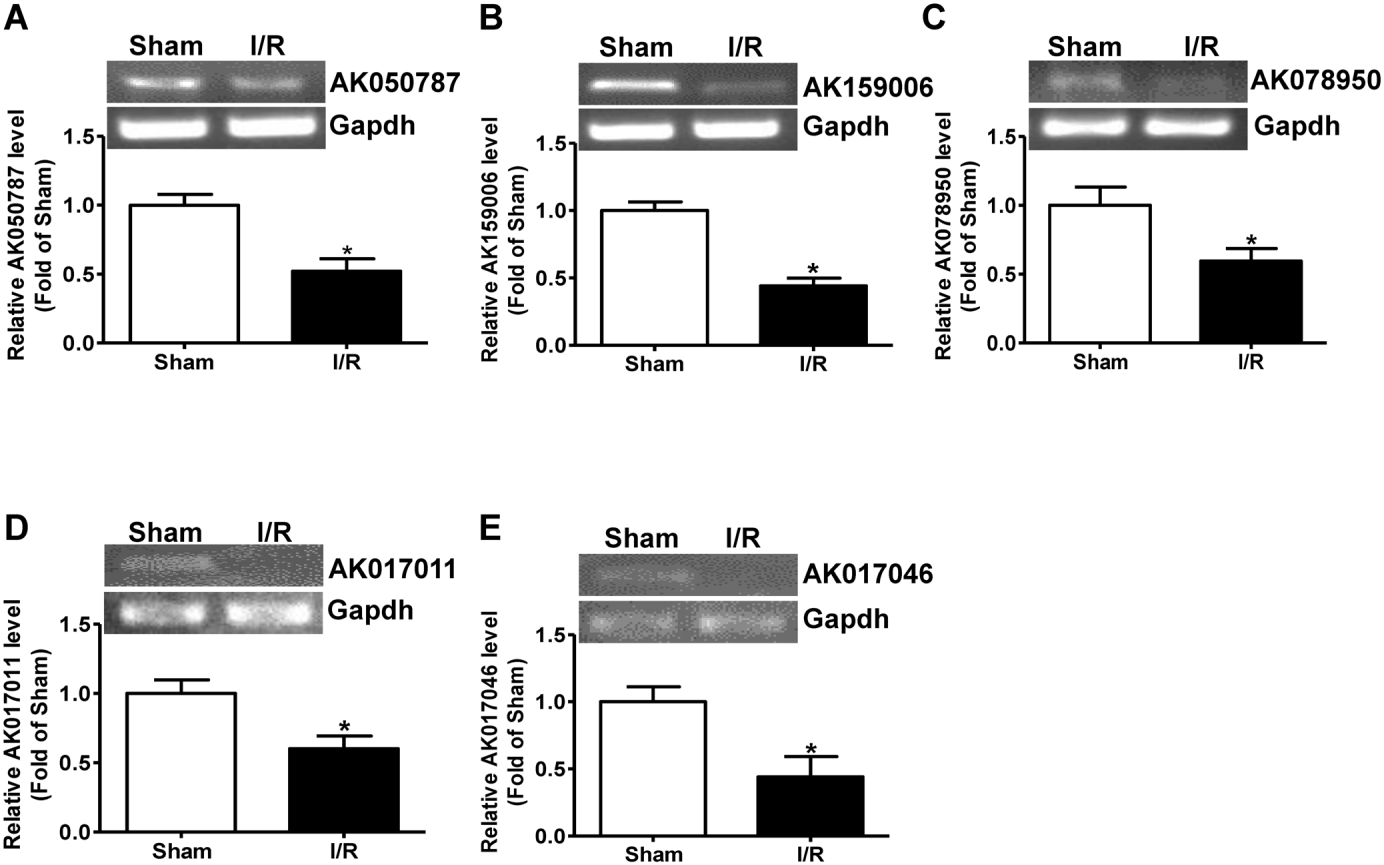
Quantitative assays of decreased LncRNAs in I/R-treated mouse plasma. A-E) Five downregulated LncRNAs with most meaningful decrease in expression fold picked up by microarray assays were further analyzed and confirmed by quantitative RT-PCR assay. In all panels, the data were normalized to sham group. The representative gel image had been provided for analysis of each LncRNA. I/R, ischemia/reperfusion; Sham, plasma of sham mice; I/R, plasma of I/R-treated mice. N = 5, *P<0.05 versus sham group.

To further validate the accuracy of LncRNA profile determined by microarray technology, some of the dysregulated plasma LncRNAs with the most meaningful change in fold (S2 and S3 Tables) were analyzed by real time PCR assays. The expression levels of five upregulated LncRNAs, AK017799, AK082383, AK042407, AK013346 and mouselincRNA0842- (S2 Table) were analyzed by real time PCR assays. AK017799, AK082383, AK013346 and mouselincRNA0842 were confirmed to be significantly increased (Fig 3). Although no statistical significance was observed due to individual variation, AK042407 also tend to be increased after liver IRI (Fig 3C). The expression levels of the 5 downregulated LncRNAs, AK050787, AK078950, AK159006, AK017011 and AK017046 (S3 Table) were analyzed by real time PCR assay. The results indicated that all of them were significantly reduced in liver IRI mouse plasma than in sham mouse plasma (Fig 4). Overall, these findings confirmed the accuracy of microarray data obtained from analysis of pooled plasma.

### Comparison analysis of dysregulated LncRNA profile in the plasma and the liver of mice after IRI

**Fig 5:**
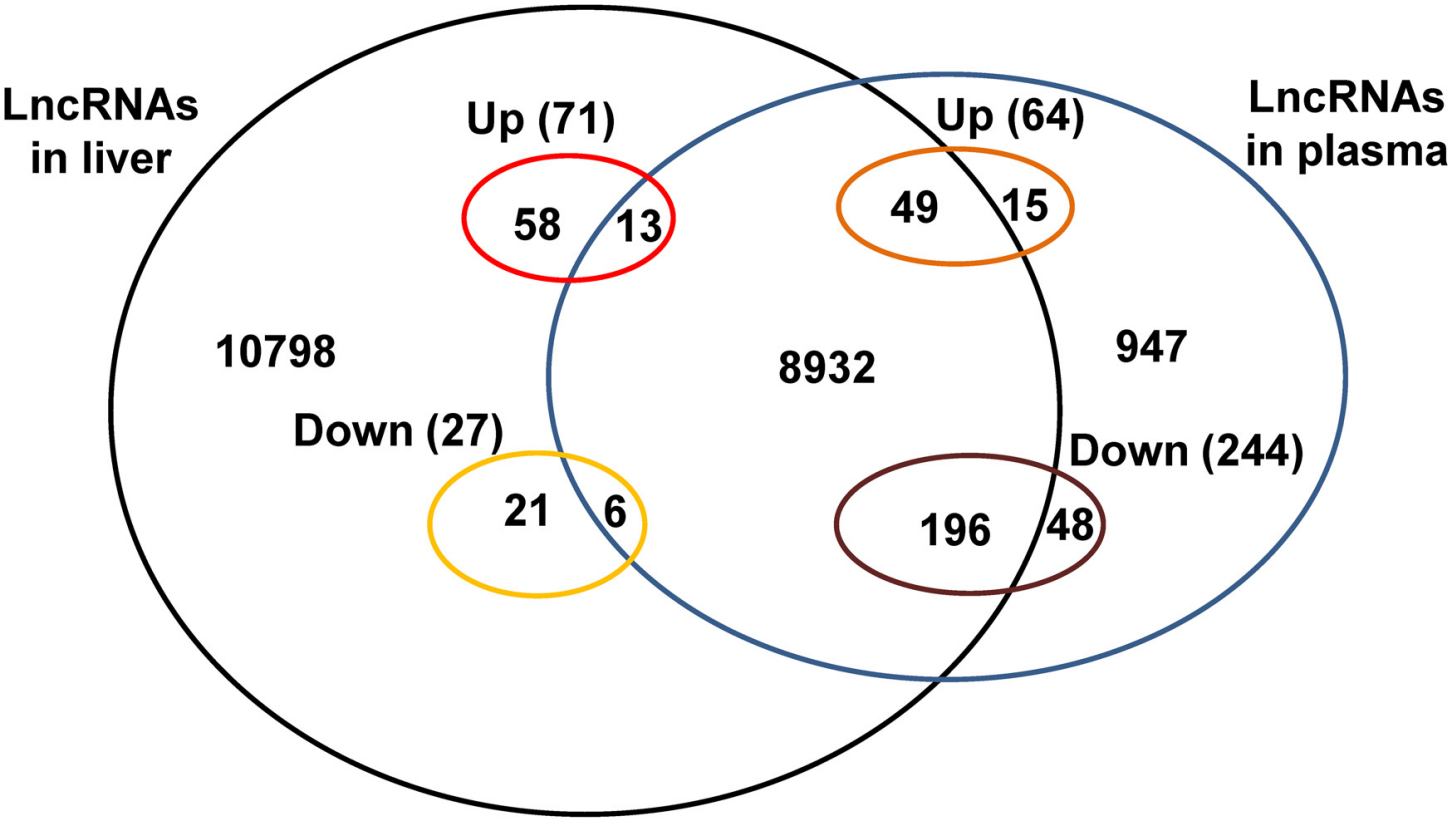
The distribution of LncRNAs between mouse liver and plasma. This figure had been made based on the data presented in Table 1, Table 2, S1 Table and LncRNA profile in the plasma and liver published in our previous study [12]. Up, upregulated LncRNAs in plasma or liver; Down, downregulated LncRNAs in plasma or liver; the numbers in the figure represent the numbers of LncRNAs present in plasma or liver. Total LncRNAs in liver: 20073 [12]; total LncRNAs in plasma: 10206; total LncRNAs present in both liver and plasma:9196.

**Fig 6:**
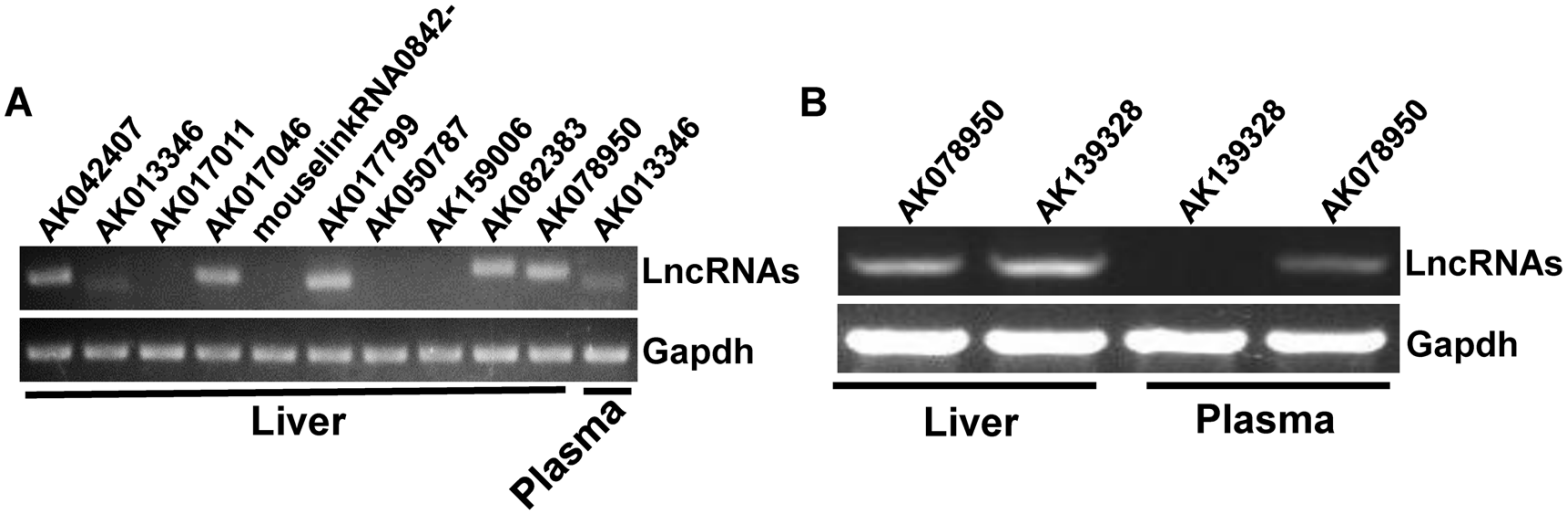
AK139328 is not present in mouse plasma. A) The distribution of validated plasma LncRNAs in mouse livers. B) AK139328 is not present in mouse plasma. RT-PCR assay was performed to detect the existence of LncRNAs in mouse plasma and livers. The gel images shown here were the representatives of 3 independent experiments.

To analyze the correlation of dysregulated LncRNAs between plasma and liver, we analyzed the expression levels of 308 dysregulated plasma LncRNAs in the liver using the microarray data published in our previous study [12]. Among the 64 upregulated plasma LncRNAs, 49 LncRNAs were detected in the liver as well, but all remained unchanged (Table 1, Fig 5), and remaining 15 LncRNAs were not present in the liver in microarray analysis data (Table 1, Fig 5). Among the 244 downregulated plasma LncRNAs, 196 LncRNAs were detected in the liver, but all remained unchanged (Table 1, Fig 5), and the remaining 48 LncRNAs were not present in the liver in microarray assays (Table 1, Fig 5). Among the 10 dysregulated LncRNAs validated by real time PCR assays, AK017799, AK082383, AK042407, AK013346, AK078950 and AK017046 were present in the liver without expression change after liver IRI. In contrast, microarray failed to detect the signals of AK050787, AK159006, AK017011 and mouselincRNA0842- in the liver (Table 2) [12]. To further confirm the accuracy of microarray data, the expression of these 10 LncRNAs in the liver was analyzed by RT-PCR. The RT-PCR assays also revealed that AK050787, AK159006, AK017011 and mouselincRNA0842- are not present in the liver (Fig 6A). In our previous study, microarray assays revealed that 98 LncRNAs were dysregulated in the liver after IRI [12]. In the current study, microarray assays revealed that only 19 of these dysregulated liver LncRNAs were present in the plasma. In contrast, microarray assays failed to detect the signals of the other 79 LncRNAs in the plasma (Table 3). Among the 11 dysregulated liver LncRNAs validated by real time PCR, microarray assays in the current study revealed the existence of AK028007, NR-028310, NR-015462 and NR-036616 in the plasma, but remained unchanged. In contrast, microarray assays failed to detect the signals of AK139328, AK087277, AK029601, AK054386, AK143693, AK143294 and ENSMUST0000151138 in the plasma (Table 4). To help clarify the correlation of dysregulated LncRNAs in plasma and liver, their distribution in liver and plasma after liver IRI had been also summarized in Fig 5.

**Table 1:** Distribution of the dysregulated plasma LncRNAs in the liver.

	Increased in plasma	Decreased in plasma
Total number	64	244
Detected in liver	49 (76.6%)	196 (80.3%)
Upregulated in liver	0	0
Downregulated in liver	0	0
Not detected in liver	15 (23.4%)	48 (19.7%)

“Not detected” means microarrays failed to detect the significant signals of LncRNAs in mouse livers. Detailed information of dysregulated plasma LncRNAs was summarized in S2 and S3 Tables. The detailed information of liver LncRNAs had been described in our previous study [12].

**Table 2:** The correlation of the validated LncRNAs between plasma and liver after ischemia/reperfusion treatment.

	Name of LncRNAs	Change in plasma	Change in liver
**Increased**	mouselincRNA0842-	2.14	-
	AK013346	1.74	0.93
	AK042407	1.69	1.17
	AK082383	1.66	0.91
	AK017799	1.65	1.02
**Decreased**	AK159006	0.44	-
	AK078950	0.47	1.11
	AK017011	0.48	-
	AK017046	0.49	0.82
	AK050787	0.53	-

“-” means no significant signal was detected in the liver using microarray technology. Detailed information of these validated plasma LncRNAs was summarized in S2 and S3 Tables. The detailed information of liver LncRNAs had been described in our previous study [12].

**Table 3:** Distribution of the dysregulated liver LncRNAs in the plasma.

	Upregulated in liver	Downregulated in liver
Total number	71	27
Detected in plasma	13 (18.3%)	6 (22.2%)
Upregulated in plasma	0	0
Downregulated in plasma	0	0
Not detected in plasma	58 (81.7%)	21 (77.8%)

“Not detected” means microarrays failed to detect the significant signals of LncRNAs in mouse plasma. Detailed information of dysregulated liver LncRNAs had been summarized in S2 and S3 Tables in our previous study [12].

**Table 4:** The correlation of the validated LncRNAs between plasma and liver after ischemia/reperfusion treatment.

	Name of LncRNAs	Change in liver	Change in plasma
**Upregulated**	AK139328	2.08	-
	AK028007	1.98	0.94
	AK054386	1.98	-
	AK087277	1.88	-
	AK029601	1.78	-
**Downregualted**	NR-028310	0.64	0.85
	AK143693	0.48	-
	NR-036616	0.49	1.10
	ENSMUST0000151138	0.60	-
	NR-015462	0.60	0.80
	AK143294	0.64	-

“-” means no significant signal was detected in the liver using microarray. Detailed information of these validated liver LncRNAs had been summarized in S2 and S3 Tables in our previous study [12].

In our previous study, we found that AK139328 is one of upregulated LncRNAs in the liver after IRI, and siRNA-mediated silencing of hepatic AK139328 significantly ameliorated liver IRI [12]. In the current study, microarray assay failed to detect the presence of AK139328 in the plasma (Table 4). High sensitive RT-PCR was further performed to confirm whether AK139328 was present in the plasma or not. RT-PCR assays also failed to detect the existence of AK139328 in the plasma (Fig 6B). AK078950, one LncRNA which is present in both plasma and liver in microarray assays (Table 2, S3 Table) [12], had been used a positive control in RT-PCR assays (Fig 6A). Overall, these findings suggested that AK139328 is likely to be an unsecretory LncRNA.

## Discussion

So far, although the functions of circulating LncRNAs still remains largely unknown, several lines of evidence have suggested that they could be novel biomarkers for diagnosis and treatment of various diseases. LncRNA uc022bqs.1, also called LIPCAR, has been reported to predict the survival of patients with heart failure [18]. LIPCAR may be a new biomarker for diagnosis of various cardiac diseases [18]. Isin and colleagues reported that several circulating LncRNAs including TUG1, LincRNA-p21, MALAT1, HOTAIR, and GAS5 could be the potential biomarkers for diagnosis of chronic lymphocytic leukemia (CLL) and multiple myeloma (MM) [19]. Arita and colleagues found that circulating LncRNA H19 level is increased patients with gastric cancer when compared with healthy subjects and reduced after surgery, suggesting that LncRNA H19 might be a new potential biomarker for diagnosis of gastric cancer [20]. Lorenzen and colleagues recently found that LncRNA TapSAKI in circulation is a predictor of mortality in critically III patients with acute kidney injury [21].

In a previous study, we found that 98 LncRNAs were dysregulated in IRI liver. Silencing AK139328 significantly ameliorated IRI of mouse liver [12]. Although our previous findings strongly suggested that dysregulated LncRNAs profile in the liver is involved in the IRI, liver LncRNAs may not be the appropriate biomarkers for diagnosis and prognosis of IRI diseases due to the difficulty in collecting human liver tissues and the ethical problems. Clearly, to determine the plasma LncRNAs profile and compare it with LncRNAs profile in the liver will be very helpful for identification of some novel biomarkers for diagnosis of liver IRI diseases.

In this study, LncRNA profile in mouse plasma after hepatic IRI was determined using microarray technology. Microarray assays detected a total of 10206 LncRNA signals in the mouse plasma (GEO accession number: GSE60726). Sixty four LncRNAs were elevated, and 244 LncRNAs were reduced in the mouse plasma after liver IRI when compared sham mouse (S2 and S3 Tables, Fig 2). About 60% of the dysregulated plasma LncRNAs are intergenic. Ten of the dysregulated LncRNAs with most meaningful change in expression fold in the plasma were further validated by real time PCR assays (Figs 3 and 4). Overall, real time PCR assays further confirmed the accuracy of microarray results. Interestingly, although LncRNA Ak013346 only yielded an increase of 1.7 fold by microarray assay (Table 2) in IRI plasma, real time RT-PCR assays detected its significant signal in IRI plasma but not in sham plasma (Fig 3E). These findings revealed the potential of AK013346 as a unique molecular marker for evaluation the severity of ischemia liver diseases. Clearly, to analyze the change in some circulating LncRNAs including AK013346 may be useful for evaluation the severity of ischemic liver damage when liver surgery is performed.

We further analyzed the distribution and expression of the dysregulated plasma LncRNAs in the liver using the microarray data of liver LncRNA profile published previously [12]. Among the 308 dysregulated plasma LncRNAs, 245 LncRNAs were found to be also present in microarray data in the liver (Table 1) [12]. Interestingly, all of these 245 LncRNAs present in the liver remained unchanged after liver IRI (Table 1). Among the 10 dysregulated plasma LncRNAs validated by real time PCR assays, 6 LncRNAs were also present in the liver but without expression change (Table 2). On the other hand, among the 98 dysregulated liver LncRNAs after IRI [12], only 19 LncRNAs were also present in the plasma (Tables 3 and 4). Clearly, these findings suggested that in IRI mouse plasma, the dysregulated plasma LncRNAs remained either unchanged (79.5%) or undetectable (20.5%) in the liver (Table 1). On the contrary, only 19.4% of the dysregulated liver LncRNAs were also detected in the plasma (Table 3). Overall, it is likely that dysregulated plasma LncRNA profile is not directly associated with dysregulated liver LncRNAs after liver IRI. In the previous study, we found that LncRNA AK139328 plays an important role in the pathogenesis of liver IRI [12]. In the current study, microarray failed to detect the signal of AK139328 in the plasma (GEO accession number: GSE60726, Table 4). High sensitive RT-PCR further confirmed that no signal of AK139328 was detected in mouse plasma (Fig 6B). This suggested that AK139328 is likely to be an unsecretory LncRNA. The number of LncRNAs identified in the cells and plasma increased rapidly in the past years [22, 23]. It is possible that further microarray assays are still needed to validate the correlation between plasma and liver LncRNA profile after liver IRI using updated LncRNA Microarray version.

Our findings also raise an important issue regarding the source of dysregulated LncRNAs during liver IRI. All dysregulated plasma LncRNAs remained either unchanged or absence in mouse livers after hepatic IRI (Table 1), strongly suggesting that the source of these dysregulated LncRNAs may not be restricted to liver cells during hepatic IRI. Our previous study had suggested that blood cells may secrete large amount of LncRNAs during heart failure [15]. However, although the expression of many LncRNAs remained unchanged in the liver after IRI, we can not preclude the possibility that their secretion was changed during IRI. It is also possible that LncRNAs could be released from necrotic liver cells during IRI. Overall, our findings suggested that the diagnosis and treatment of liver IRI diseases should not be restricted only to the liver, and the role of other tissues or blood cells should be considered.

The further study of the nature, origin and function of circulating LncRNAs will definitely shed light on the mechanisms of many diseases such cancer, IRI and metabolic diseases. In particular, to study the mechanism of LncRNA secretion from various cell types will also be important for understanding its biological function under physiological and pathophysiological conditions. Overall, further study on circulating LncRNAs and their correlation with various diseases will be useful for identification of novel biomarkers for diagnosis and targets for treatment of diseases.

In conclusion, the correlation between dysregulated LncRNAs profile in plasma and liver after hepatic IRI was comparably analyzed. A total of 308 LncRNAs were dysregulated in the plasma of mice after hepatic IRI. All the dysregulated plasma LncRNAs remained either unchanged or undetectable in the liver. On the other hand, all dysregulated liver LncRNAs also remained either unchanged or undetectable. Clearly, during liver IRI, the source of dysregulated plasma LncRNAs is not restricted to liver cells. Analyzing the change in some circulating LncRNA levels may be useful for evaluating the severity of ischemic liver diseases.

## Supporting Information

S1 Table List of oligonucleotide primer pairs used in real time RT-PCR and RT-PCR analysis. (DOC)

S2 Table The characterics of increased LncRNAs in Ischemia/Reperfusion injured mouse plasma. (XLS)

S3 Table The characterics of decreased LncRNAs in Ischemia/Reperfusion injured mouse plasma. (XLS)
