# Supplementary material for: Comparison Analysis of Dysregulated LncRNA Profile in Mouse Plasma and Liver after Hepatic Ischemia/Reperfusion Injury
Source: PLoS One. 2015 Jul 29;10(7):e0133462. doi: 10.1371/journal.pone.0133462 (PMC4519261; doi:10.1371/journal.pone.0133462)
Supplement: S1 Table — (DOC) [file pone.0133462.s001.doc]

**Table S1. List of oligonucleotide primer pairs used in real time RT-PCR and RT-PCR analysis**.

| Target Gene | Sense Primer(5'-3') | Antisense Primer(5'-3') | Annealing temperature |
| --- | --- | --- | --- |
| AK013346 (M) | AGGAGAGTTTGCTCAACCCTT | TGTGCACCCCACTCACTAAC | 59℃ |
| AK017799 (M) | AATGTGTGGGTGTGATGCCT | GCCATGTCTGACTCCTGTCC | 59℃ |
| AK042407 (M) | GTACCATTTGGCTTCTGCGG | AGCCTGGCTATTCCACCTCT | 60℃ |
| AK082383 (M) | TACTCTCTTGGCCACACTGC | TAGCGTTACTGTGCTGGCTC | 60℃ |
| MouselincRNA0842- | CCTCATCCTTGTCCAGCCTA | AGTGGAGGTCTCAGGAGCAA | 59℃ |
| AK017011(M) | aaaccaggctgtccaagatg | cagcatccctcagtcctctc | 58℃ |
| AK017046 (M) | GGCTCGGTGACTAAGAGCAC | AGCACCGGGATTTTGTGTGT | 57℃ |
| AK050787 (M) | agggtcgagaaggagagagg | aggacctgaagtgctctgga | 59℃ |
| AK078950 (M) | TTTCCGAGGTTCCCTCTCCT | GAGTGTGACCTTCCGGCTAC | 57℃ |
| AK159006 (M) | TCCAATGCAATGTCGGGGAA | TGCACAGAGTGAGCTCCAAG | 59℃ |
| GAPDH (M) | aactttggcattgtggaagg | acacattgggggtaggaaca | 59℃ |

M: mouse; If not indicated, all the primer sequences are referred to mouse plasma.
